# Maternal caretaking behavior towards a dead juvenile in a wild, multi-level primate society

**DOI:** 10.1038/s41598-022-08660-9

**Published:** 2022-03-21

**Authors:** Bin Yang, James R. Anderson, Min Mao, Kaifeng Wang, Baoguo Li

**Affiliations:** 1 Shaanxi Key Laboratory for Animal Conservation, College of Life Sciences, Northwest University, Xian, 710069 Shaanxi China; 2 Shaanxi Key Laboratory for Animal Conservation, Shaanxi Institute of Zoology, Shaanxi Academy of Sciences, Xian, 710032 Shaanxi China; 3 Department of Psychology, Kyoto University Graduate School of Letters, Kyoto, 606-8501 Japan; 4 Department of Management and Marketing, School of Business and Economics, Universiti Putra Malaysia, 43400UPM, Serdang, Selangor Malaysia; 5 Center for Excellence in Animal Evolution and Genetics, Chinese Academy of Sciences, Kunming, 650223 China

**Keywords:** Psychology, Zoology

## Abstract

Maternal caretaking and transport of dead infants are widespread among nonhuman primates, having been reported in numerous species of monkeys and apes. By contrast, accounts of such behaviors toward dead juveniles are scarce. Here, we describe responses by the mother and other group members to the death of a juvenile in a wild, multi-level group of Sichuan snub-nosed monkeys (*Rhinopithecus roxellana*). Following the juvenile’s fatal accident, his mother transported and cared for the corpse for four days. Immature monkeys belonging to the same one-male unit, and some individuals from other social units also showed interest in and tended the corpse. Comparisons of this case with those involving the deaths of infants and an adult female in the same population highlight possible effects of physiological, psychological and emotional factors in primate thanatological responses, and provide an additional perspective on the origin and evolution of compassionate acts.

## Introduction

Responses to dying and dead conspecifics across animal species reveal a continuum ranging from hard-wired fixed-action patterns to varied and flexible behaviors with cognitive and emotional correlates^1^. Describing and studying these responses can clarify adaptive and possible maladaptive behaviors in various species, as well as the underlying biological (including psychological) mechanisms in death-related responses^1–3^. The comparative thanatology literature includes many reports of responses toward corpses in nonhuman primates: prosimians, monkeys and apes^2–4^. In particular, care and transport of dead infants by their mother and sometimes other individuals has been described in a range of primate species including, among others, capuchin monkeys (*Sapajus* sp.)^5^, macaques (*Macaca* sp.)^6,7^, baboons (*Papio* sp.)^8,9^, geladas (*Theropithecus gelada*)^10^, chimpanzees (*Pan troglodytes*)^11–15^, and mountain gorillas (*Gorilla beringei beringei*)^15,16^. Based on behaviors such as social withdrawal and reduced interest in the environment, many authors have suggested that mothers continuing to care for and look after their lifeless infants are experiencing grief. The mechanisms underlying these behaviors are still debated, but the interaction of maternal physiological and emotional factors, including the strength of the bond with the offspring seems highly likely to be involved^14,17,18^.

Although the phenomenon of mothers continuing to carry and care for dead infants is widespread among anthropoid primates, accounts of such responses to dead juveniles are notably lacking. For example, researchers studying Yunnan snub-nosed monkeys (*Rhinopithecus bieti*)^19^ and Sichuan snub-nosed monkeys (*Rhinopithecus roxellana*)^20,21^ reported carrying and care of dead infants, but not juveniles, and dead infant geladas (*Theropithecus gelada*) received more attention than dead juveniles and adults^10^. However, in chimpanzees, dead adults and subadults may receive more attention than dead infants^22^, although the larger corpses are not carried. Mortality in infant primates is much higher than in juveniles, which can at least partly explain the prevalence of accounts of infant deaths and postmortem care. More descriptions of responses to dead juveniles are needed, to compare and contrast responses to corpses belonging to different age categories across species.

Here, we report the first known case of maternal transport and caretaking of a dead juvenile, along with responses by other individuals, in a wild group of Sichuan snub-nosed monkeys. These monkeys live in temperate forests on mountain plateaux in central and southwestern China, at 1500–3400 m. They are described as living in a multi-level society, consisting of several one-male units (OMU) and associated all-male units (AMU)^23–27^. Overall, the group has 50–150 individuals, with each OMU having a single resident adult male, several adult and sub-adult females, juveniles and infants^24–30^. Within this society the monkeys have multiple levels of social interactions and relationships^23–27^. Although most affiliative behaviors occur within OMUs, infants and juveniles often visit other units and form play groups of 10–30 individuals; other behaviors such as grooming and co-feeding may also occur^23–27^. Endemic to China, Sichuan snub-nosed monkeys are seasonal breeders, in which mating occurs from September to December, and births occur from March to May^21,28–30^.

Newborn infant snub-nosed monkeys are at risk of dying from cold weather, congenital abnormality or disease, and other, less common causes such as accidents or infanticide^20,21,31^. To contextualize the responses observed toward the dead juvenile described here, we compare and contrast this case with previously described responses toward dead infants and and an adult female in the same population^20,21,32^. This account highlights the multiplicity of factors involved in primate thanatological behaviors. It thus contributes further evidence for variability in primate reactions, and to the debate surrounding death-related compassion and grieving in nonhuman species^18,32–36^.

## Methods

Observations were conducted on Sichuan snub-nosed monkeys on the southern slopes of the Qinling Mountains near Longcaoping Village in Guanyinshan National Nature Reserve (107°51′-108°01′E, 33°35′-33°45′N), Shaanxi province, central China^28–30^. The study group has been habituated to researchers since 2010 and can be observed at close range, with individual recognition based on various physical characteristics^28–30^. The monkeys were categorized into seven age/sex classes as described in previous studies^23,24,28^: adult males (>7 years old), adult females (>5 years old), subadult males (5–7 years old), subadult females (3–4 years old), juvenile males (1–4 years old), juvenile female (1–3 years old), and infants (3 months to 1-year-old).

The study group consisted of 92 individuals organized in seven one-male units (OMUs) and one associated all-male unit (AMU) in June 2016. The OMU in which the death occurred contained an adult male (*SBW*, three adult females (AF1: *CM*, AF2: *XW*, AF3: *JD*), five juveniles (J1, J2, J3, J4, and CM’s offspring: *CM-J*) and one infant (JD-I). We recorded the reaction of the OMU members to the dying juvenile using focal-animal, ad-libitum sampling^37^. We observed through binoculars when necessary (Nikon 7245 Action Ex Extreme 10 X 50 mm), recorded times with a stopwatch (Casio Men’s AE1000W-1B, wrote behaviors in a notebook, and took photographs (Canon cameras EOS-1D X, EOS 700D, and EOS-5D Mark II). The death occurred at the Daping valley (107°59′6.5688"E, 33°40′19.0164"N), well inside the group’s home range. The study adhered to the legal requirements of the Guanyinshan National Nature Reserve, China, and the guidelines of the International Primatological Society for the ethical treatment of primates. The study was approved by the animal care committee of the Wildlife Protection Society of Shaanxi Province, China (permit number: SX43537ACC).

## Results

Here, we present the main events observed over a 5-day period covering the juvenile’s death, maternal care and transport of the corpse, and ending on the day following abandonment of the corpse. Based on the ad-libitum observations, Table 1 summarizes selected behaviors of the mother before the death of the juvenile and during the days when she carried and cared for the corpse. Table 2 summarizes other group members’ responses to the juvenile before and after his death, and after abandonment by the mother, and Table 3 summarizes their responses to the mother. The descriptions below focus on some of the most notable events observed. A comprehensive photo collage with a more complete timeline including all behavioral descriptions extracted from notes and photos is presented as online supplementary material.

**Table 1:** Comparison of mother’s behavior before and after the day that CM-J died.

Behavior	Before CM-J dead	After CM-J dead
Alarm calling	No occurrence	Frequent
Crying contact calling	No occurrence	Frequent
Sitting beside	Other OMU females	Mostly with CM-J
Looking	No clear pattern	Mostly toward CM-J
Sniffing another’s face	No occurrence	Directed toward CM-J
When immatures approached	Remained neutral	Some threat responses
Grooming bouts	No preferred partner	Focused on CM-J
Spatially separated from OMU	No occurrence	Separated, remains with CM-J
Approaches	No preferred partner	Focused on CM-J
Carry	Seldom	Frequent, focused on CM-J
Prolonged, passive sitting in silence	No occurrence	Beside CM-J

**Table 2:** Responses to the juvenile (alive and dead) by group members.

	Age and sex	Before dead			After dead			After abandonment		
		Grooming	Proximity	Others	Grooming	Proximity	Others	Grooming	Proximity	Others
Intra-unit	AM	Rare	Normal	None	None	Rare	Rare; Look	None	None	None
	Mother	Less	Normal	None	Frequent	Frequent	Frequent: Inv, Look, Aff	None	None	Rare; look, plaintive contact call
	AF	Rare	Normal	None	Rare	Rare	Rare: Inv, Look, Aff	None	None	None
	Imm	Less	Frequent	Frequent play	Rare	Rare	Less: Inv, Look, Aff	None	None	Rare: look, alarm calls
Extra-unit	M	None	None	None	None	None	Rare: Look	None	None	None
	AF	None	None	None	None	None	Rare: Look	None	None	None
	Imm	Less	Frequent	Frequent play	Rare	Few	Less: Inv, Look, Aff	None	None	Rare: look, alarm calls

AM: Adult-male, Mother: CM, AF: Adult-female, Imm: Immature (infant or juvenile);

Inv: Investigate: includes looking closely, sniffing, briefly touching; Look: visual orientation toward target individual but with no close approach; Aff: affiliative behavior including grooming, embracing, or gentle manipulation.

**Table 3:** Response to mother by group members.

	Age and sex	Before dead			After dead			After abandonment		
		Grooming	Proximity	Others	Grooming	Proximity	Others	Grooming	Proximity	Others
Intra-unit	M	Normal	Normal	None	Rare	Rare	Rare: Look	Normal	Normal	None
	AF	Normal	Normal	None	Rare	Rare	Rare: Look	Normal	Normal	None
	Imm	Rare	Normal	None	None	Rare	Rare: Look	Rare	Normal	None
Extra-unit	M	None	None	None	None	None	Rare: Look	None	None	None
	AF	None	None	None	None	None	Rare: Look	None	None	None
	Imm	None	Rare	None	None	Rare	Rare: Look	None	Rare	None

AM: Adult-male, AF: Adult-female, Imm: Immature (infant or juvenile);

2, Inv: Investigate, includes looking closely, sniffing, briefly touching; Look: visual orientation toward target individual but with no close approach; Aff: affiliative behavior including grooming, embracing, or gentle manipulation.

### Day 1, 28 June 2016: fatal accident and initial reactions

**Figure 1:**
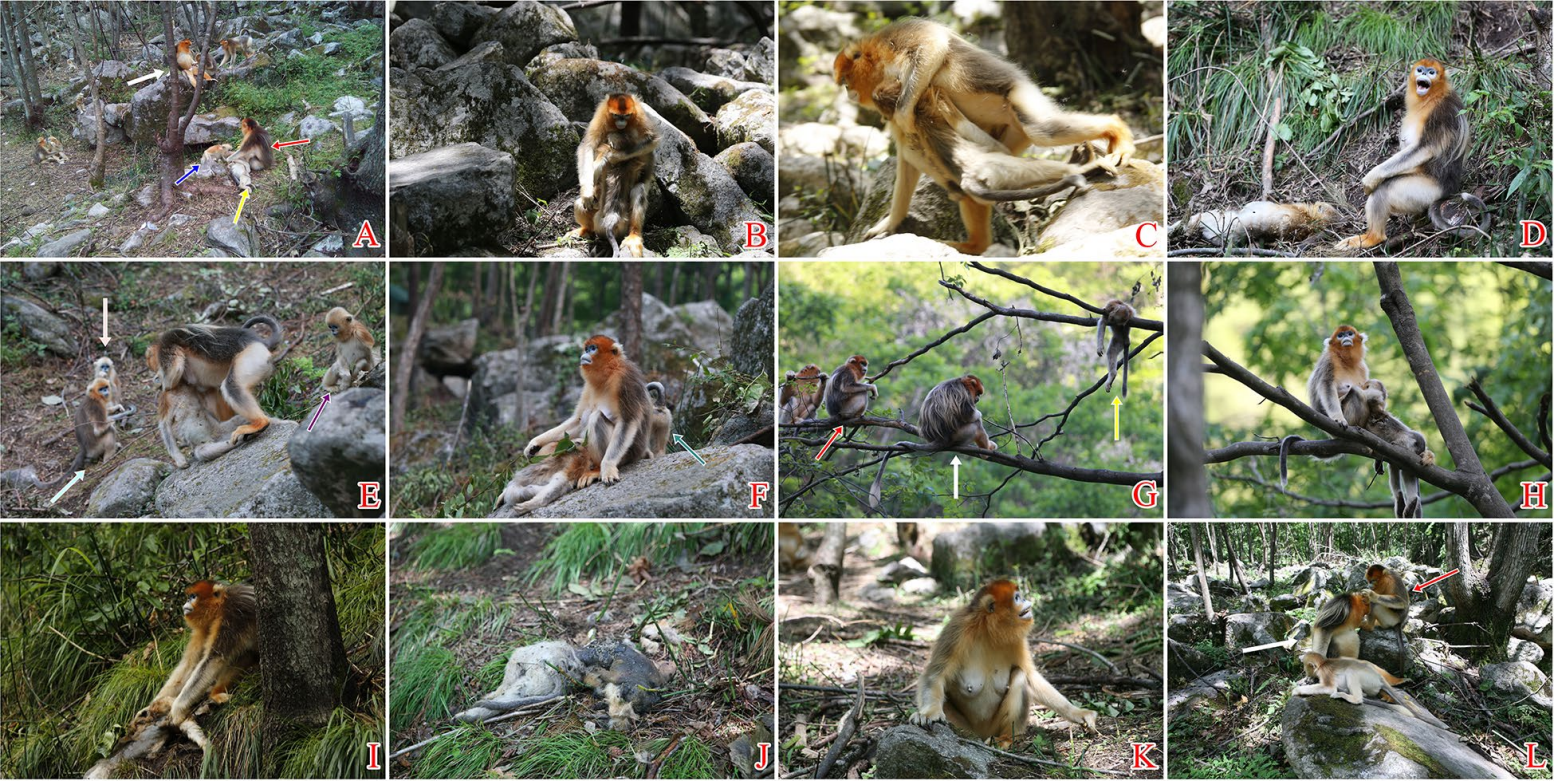
Responses of group members in *Rhinopithecus roxellana*. (**A**) The mother (red arrow) holds the dead juvenile (yellow arrow), and juvenile J3 (blue arrow) touches it. The OMU adult male (white arrow) remains nearby in a tree. (**B**) The mother holds the head of the corpse against her chest. (**C**) The mother struggles to walk with the corpse. (**D**) The mother, sitting alone beside the dead juvenile, emits calls. (**E**) The mother carries the corpse as she moves towards her OMU. (**F**) After the mother laid some leaves on the corpse a juvenile (teal arrow) approached, looked and left. (**G**) The mother (red arrow) draped the corpse (yellow arrow) over a branch, and moved to sit near the adult male (white arrow). (**H**) The mother put the head of the dead juvenile against her left breast, as if breastfeeding. (**I**) The mother had difficulty carrying the now-clearly decomposing corpse. (**J**) The decomposing body of the dead juvenile. (**K**) The mother looks toward where she abandoned the corpse and emits contact vocalizations. (**L**) The mother (red arrow) grooms the adult male (white arrow).

At 06:12, the juvenile *CM-J*, about 18 months old, was playing with another juvenile (J3) when he fell about 20 m from near the top of a tree, striking his head on a large rock as he hit the ground and apparently dying instantly. Figure 1A shows some of the behaviors seen during the next few minutes. *CM-J*’s mother (*CM*) immediately ran over, held and inspected the inert juvenile, and then mouthed and groomed him, while intermittently emitting occasional alarm calls. Juvenile J3 climbed down the tree, then approached, looked at, and lightly touched *CM-J*; he then gently mouthed *CM-J*’s face, and groomed the body. Other members of the OMU looked on from slightly farther away. Adult female *JD* stopped grooming *SBW* (the presumed father of *CM-J*), who yawned rapidly in succession while sitting on a tree branch above the dead juvenile. Another adult female (*XW*) arrived, and approached *SBW*. Juveniles J1 and J2 watched *CM* and the corpse from slightly farther away, embracing each other. *JD* resumed grooming *SBW*, and *XW* moved closer to them. Social grooming among other OMU members farther away appeared exaggerated, with some individuals appearing anxious or disconcerted.

Over the next two hours, *CM* alternated between sitting silently by *CM-J*’s corpse, and grooming it. At 08:19 she picked it up, carried it for about 30 m, then placed it on the ground and started feeding. At 10:01, she carried it into a tree where she rested, and groomed it. At 12:59 she carried it down from the tree, and at 13:01, gripping its shoulder with one hand she brought its face against her chest (Fig. 1B). At 13:03, she turned the corpse to face away from her, and held it against her chest with one hand as she clumsily walked forward. At 13:07 she again brought its face against her chest, and struggled to walk while clasping the corpse (Fig. 1C), before laying it on the ground and feeding. At 14:52, as *CM* fed about 2 m away from the corpse, an extra-unit juvenile touched and groomed it. An extra-unit infant approached, looked at, touched and briefly groomed it, then left. At 15:05, *CM* carried the corpse into a nearby tree, and alternated between grooming it and sitting motionless.

At 17:05 the group started to leave the area and move up into the mountain toward a sleeping site. *CM* carried the corpse down from the tree in which she had been resting. At 17:09, she held the corpse (facing outward) against her chest with both hands, and emitted “Wa Wa Wa” calls. A juvenile from another OMU approached and looked at the mother-corpse pair. Starting at 17:10 and for approximately 7 min *CM* alternated between holding the corpse to her chest with one hand and dragging it along the ground as she walked, and placing it on the ground. She then put the corpse on the ground and vocalized, possibly in response to people nearby (Fig. 1D).

At 17:17 she put the corpse on the ground, and was left alone as other monkeys moved farther away. *SBW* looked and called towards *CM*, who simply looked back at him. The entire group appeared to move more slowly than normal, and their sleeping site for this night was less far up the mountain than normal.

### Day 2, 29 June: postmortem care and transport

At 06:10 *CM*, lagging behind the group, put *CM-J*’s corpse on the ground as the other members of the group continued to move farther away. At 06:14 *CM* carried the corpse toward her OMU; a juvenile approached and watched from a tree. At 06:19 *CM* again carried the corpse nearer the OMU (Fig. 1E). Extra-OMU juveniles (teal arrow, cream arrow) approached and/or looked, while an extra-unit adult female also watched (light blue arrow).

At 06:20, *CM* laid the corpse down on the large stone on which she was sitting. She picked up a few leaves from the ground and placed them on the corpse’s back. A juvenile approached (Fig. 1F), looked, then left. At 06:21 *CM* picked up the corpse, carried it over approximately 5 m, and then put it down again; an infant from her unit approached, looked and then left. *CM*, now sitting alone with the corpse, occasionally vocalized. At 06:45 she picked it up and carried it into a tree, with extra-unit females looking on. At 06:50 *CM*, sitting on a branch, partially draped the dead juvenile across her lap. At 07:01, she draped the corpse over a branch and left it there as she moved to sit near *SBW* (Fig. 1G); there she ate leaves until 07:29, occasionally looking back at the corpse At 7:30, having retrieved the corpse, *CM* brought its head to one of her nipples, as if breastfeeding (Fig. 1H). For most of the next hour she held and occasionally groomed the corpse, interspersed with periods of simply gazing into space.

At 08:27 *CM* descended with the corpse and left it on the ground while she fed nearby. Infants and juveniles from *SBW*’s and other units sporadically approached, looked at, touched or groomed it, and then left. *CM* continued to emit occasional alarm calls. At 09:35 she carried the corpse into a tree, groomed it, and rested. At 14:25 she descended and laid the corpse on the ground to feed nearby; other individuals generally behaved as previously. At 16:01 *CM* picked up the corpse, carried it a short distance, then sat and groomed it. At 17:18, she carried it while following the rest of the group in the direction of the mountain.

### Day 3, 30 June: continued caretaking of decomposing corpse

At 06:01 the group arrived at the feeding site, but *CM* did not appear until 06:15, holding the corpse, which she occasionally left on the ground as she walked around or rested. Near her OMU at 06:45, she carried the corpse into a tree, and held it while she sat alone, variously grooming, mouthing, inspecting and touching it, and resting. At 08:21, *CM* descended from the tree, placed the corpse on the ground, and ate alone nearby. Between 08:38 and 09:49 four intra-unit and four extra-unit youngsters (infants or juveniles) individually approached and touched, mouthed, and groomed it before leaving; *CM* occasionally gave alarm calls. At 10:01 *CM* carried the corpse into a tree and held it as she rested, occasionally grooming it.

At 14:38 *CM* descended, placed the corpse on the ground, and ate alone nearby. Between 14:51 and 16:15 five other-unit and four same-unit youngsters individually approached and touched, mouthed or groomed the corpse, with *CM* again occasionally alarm calling. At 16:21 *CM* carried the corpse to a large rock, where she sat alone and groomed it. At 16:38 adult female *XW* approached, briefly mouthed it, and left.

At 17:15, the group started to move up the mountain for the night. *CM* tried to follow, dragging the corpse along the ground. By 17:21 the group was out of view, with *CM* lagging behind. The corpse was now clearly decaying, with large patches of fur missing. At 17:21 *CM* again tried to follow the group while holding the corpse, but at 17:21 she stopped, put it on the ground, and looked in the direction where the group had gone (Fig. 1I). As on the previous day, the group moved notably slower than usual; this was especially true for *CM*’s unit.

### Day 4, 1 July: abandonment

At 11:06, having eaten, the group was resting in the feeding area. However, *CM* was still on the road approximately half-way up the mountain. As she moved she tried repeatedly in vain to pull *CM-J*’s corpse, which had further decomposed; it had lost more fur, some of which was scattered across the ground, its exposed flesh wet and slippery, and emitting a pungent odor. *CM* sat beside it, then moved to rest in a nearby tree, where she self-groomed. The now almost furless corpse was infested by maggots, and the head was unrecognizable (Fig. 1J). At 11:27 *CM* continued sitting in the tree and self-groomed, occasionally looking at the corpse and in the direction of the group. Shortly thereafter she rejoined her unit, leaving the corpse where it was.

At 11:56 *CM* sat next to *SBW*, with *XW* alongside. At 11:57 she ate while sitting beside the male. At 12:00 and still feeding, *CM* occasionally looked in the direction of the hillside (out of view) where she had left the corpse, and occasionally uttered the plaintive contact call “A wo wo” (Fig. 1K). This is a different kind of sound from the usual contact sound made while foraging, typically used when an individual gets separated from the group or searches for a specific individual. At 12:10 *CM* approached and groomed *SBW*, while a juvenile groomed *CM*. At 12:18 she sat alone while the other members of her unit fed. She stood up and walked to a large nearby boulder and sat down again, facing and occasionally looking in the direction of the hillside as before; she sometimes self-groomed. At 12:23 *CM* joined *SBW* and groomed him (Fig. 1L).

At 12:56, due to the unpleasant odour and concerns about possible disease risk, a field assistant started to bury *CM-J*’s remains. Some monkeys became aware of the burial, approached (*CM* was not among them), and occasionally alarm called from up in the trees. Later that afternoon, *CM* groomed with other members of her unit. At 17:00, the group moved up the mountain, notably faster than the previous day, and covered a greater distance to reach their sleeping site.

### Day 5, 2 July: post-abandonment

The group’s overall behavior appeared normal; in particular, *CM*’s locomotion, feeding and socializing appeared unremarkable, including grooming and being groomed by her unit’s females. However, she occasionally emitted plaintive contact calls; for example, at 12:40 when, after eating alone, she emitted a series of: “A wo wo” vocalizations.

**Figure 2:**
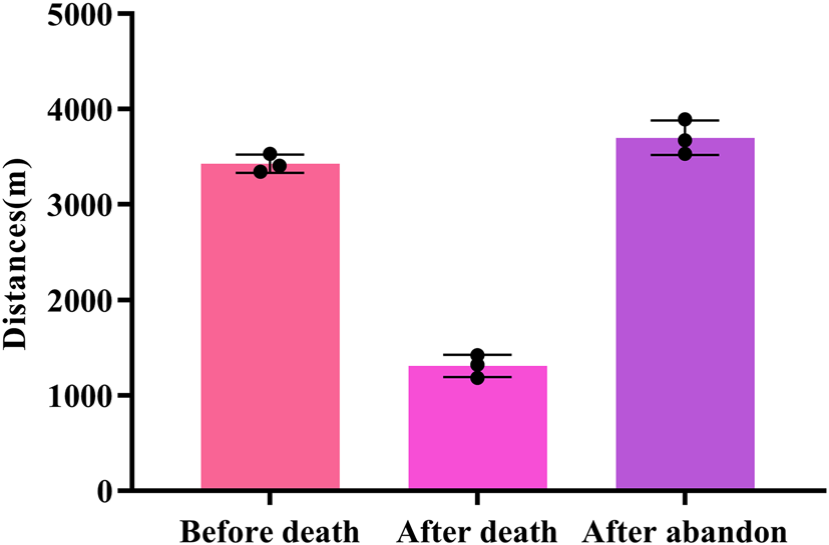
The average distances covered by the group and mother before, the juvenile's death, after death, and after corpse abandonment by the mother.

The distance traveled by the mother and the group was calculated for the 3 days before *CM-J*’s death (mean: 3,427.0 m; SD = 95.9 m), the 3 days following the death (mean: 1,310.7 m, SD = 117.0 m), and the 3 days after *CM* abandoned the corpse (mean: 3,699.3 m, SD = 182.5 m) (Fig. 2). These distances reflect overall slower movement and and reduced ranging by the group during the days when the mother was carrying the juvenile’s corpse, compared to the pre-death and post-abandonment periods.

## Discussion

Reports of responses to dead conspecifics in primates indicate wide variability, with the setting, cause and context of death, age and sex of the dead individual and the nature of the social relationships between the latter individual and other group members all potentially likely to influence those responses^2,38^. The most striking feature of the present case, the first concerning a dead juvenile Sichuan snub-nosed monkey, was the mother’s heavy investment in carrying and caring for her juvenile’s corpse; she was in physical contact with or proximity to the corpse before abandoning it on the fourth day after death. Despite the corpse being considerably larger and heavier than that of an infant, several of the mothers’ actions toward the corpse—carrying, peering, gently mouthing face, grooming, embracing and gentle pulling—are highly similar to those reported in mothers of dead infants, and strongly suggest a continuing emotional attachment. Furthermore, the mother periodically gave alarm calls, usually emitted in response to some kind of danger such as the approach of a potential predator, suggesting protectiveness.

Although she was clearly protective of the corpse, the mother did allow infants from her unit and other immatures to approach and contact it (Table 2). Mothers in other primate species are reported to tolerate some close inspection and manipulation of their dead infant by others, especially kin, but they also spend more time alone—perhaps partly due to others avoiding the smell of the decaying corpse^7^—and may reject some approaches and attempts to contact it^6,7,9,18,39,40^. Intra- and inter-species variability in maternal tolerance of access to dead offspring by other members of the group remains to be more thoroughly studied.

The overall demeanor of the bereaved mother was consistent with being in a state of grief, consistent with other reports of bereavement in primates^11,14,33–36^. For long periods she simply sat alone with the corpse, in silence but for occasional contact vocalizations usually made while searching for a missing individual. Furthermore, in contrast to most mothers with a live offspring, she generally avoided other individuals; she was less often in proximity to them and received less grooming than before the death. The amount of grooming received by the mother recovered after she abandoned the corpse, similar to but faster than in mothers with dead infants^20^. This might suggest disruption to social interactions due to the mere physical presence of the corpse, or simply a faster recovery from any grief response to the loss of a juvenile compared to an infant; further data are required to clarify this issue. Shortly after finally abandoning the corpse, she repeatedly looked back to where she had left it, emitting contact calls. However, by the following day her behavior appeared to have returned to normal in terms of movement, feeding, and social interactions. We have no physiological data covering the period of this study, which is unfortunate as maternal hormonal status can provide valuable information about the bereaved mother’s emotional state^41,42^_._

Some of the mother’s behaviors were untypical of mothers with live offspring, such as placing leaves on the corpse, and manually bringing its head to her nipple. The function of the former, if any, is unclear, but it might be similar to Taï chimpanzees’ behavior of dropping vegetation onto some corpses, perhaps testing for a reaction^43^. Holding the dead juvenile’s face against her nipple recalls the observation of an adult female Hanuman langur with a dead 8-month-old infant sucking her nipple until milk was expressed and then bringing the infant’s head into contact with the nipple^44^. These acts appear to be attempts by these mothers to elicit suckling, which would suggest that they did not fully understand that the juvenile was dead, or that their understanding of death lacked the non-functionality and/or irreversibility subcomponents of the death concept typical of humans^1,3,38^. Additonally, the continued contact calling by the mother even after she abandoned the corpse might also indicate lack of its non-functionality and irreversible condition.

In contrast to some of the younger monkeys, adults generally showed no increased affiliative behavior toward the bereaved mother while she carried the corpse (Table 3), as well as relatively little interest in the corpse. However, they travelled more slowly than normal and occasionally vocalized towards the mother, suggesting that there was not total indifference toward the mother-dead juvenile pair. Nonetheless, this general lack of interest contrasts with interest and affiliation towards a dying adult female seen in other adults of the same OMU in this population of Sichuan snub-nosed monkeys^32^. In that case, the OMU adult male was particularly strongly bonded with the female, and for around 10 min after she died he remained with her corpse, gently manipulating and pulling at it. He abandoned the corpse hesitatingly, and gazed alternately between the corpse and the rest of the group before finally leaving.

Starting at about 3 months of age, infant snub-nosed monkeys can leave their mother and unit to form play groups with immature members of other family units^23–26^. By about 10–12 months age they are almost fully independent: most are weaned, they spend more time playing with unit and extra-unit juveniles and infants, and are carried and groomed by the mother much less than before^23–26^. Presumably, infants and other juveniles in the group had become friends through growing up and playing with the *CM-J* before his fatal accident, and so were naturally curious about what had happened to their companion. By approaching and engaging with the corpse youngsters may learn about death. Older individuals, by contrast, are more likely to have already experienced death events and so are less curious about it^34,39–41,45,46^; hence the overall lack of interest shown in the corpse by most adults. The strength of the mother’s bond with her juvenile, however, caused her to continue transporting and caring for him even after his death.

Previous reports of responses to dead newborn infants in snub-nosed monkeys indicate that interactions with the corpse were restricted to only the mother and siblings^20,21^. By contrast, several immature individuals with close social ties with *CM-J* when he was alive were seen to gently contact his corpse. These approaches continued up until the mother abandoned the corpse, after which they ceased, but some alarm calls continued to be emitted, possibly also reflecting the influence of social bonds.

Climate may influence corpse-carrying behavior^10^. The death-related events described here took place during the summer, when the combination of rapid decomposition, flies, and maggots invading the increasingly naked and wet corpse conceivably hastened its abandonment by the mother. Systematic comparisons of reactions to corpses at different times of year are required to better understand the influence of climatic factors on responses to corpses.

In human societies, even genetically unrelated people may care for sick or dying others, especially if they are socially close. What appears clear from the accumulating comparative literature, including the present report, is that compassionate caretaking is unlikely to be unique to humans; at least some elements may be found in other primates. Although we need to guard against anthropomorphism when interpreting reactions other species^47^, arguably especially those living in multi-level societies, we can conclude that deaths in those species appears to activate some of the behavioral and affective mechanisms that underlie the varied and complex thanatological practices that characterize human societies. Case-studies that are largely descriptive, such as the present one, are important for building a more complete picture. Where possible, however, more quantitative data are desirable: multiple variables including social interactions, other behavioral activities, age-sex class of dead individuals and surviving kin, social status, maternal parity, and climatic factors are all of interest. Supplemented with behavioral and physiological measures, such information will help to further develop the growing field of comparative thanatology, clarifying other species’ conceptions of death.

## Supplementary Information

Supplementary Information 1.

Supplementary Information 2.
