# Supplementary material for: Maternal caretaking behavior towards a dead juvenile in a wild, multi-level primate society
Source: Sci Rep. 2022 Mar 21;12:4780. doi: 10.1038/s41598-022-08660-9 (PMC8938436; doi:10.1038/s41598-022-08660-9)
Supplement: Supplementary file 1 — Supplementary Information 1. [file 41598_2022_8660_MOESM1_ESM.docx]

**Supplementary Material: Figure Legend**

**Fig. 1.** Responses of group members to the death of a juvenile *Rhinopithecus roxellana*.

A: 06:12: The mother (Red arrow) holds the dead juvenile (Yellow arrow), and emits alarm calls (“Wuga”). Juvenile J3 (Blue arrow), who was playing with CM-J has rapidly descended from the tree and touches the corpse. Adult male and OMU leader, SBW (White arrow), watches from a tree above, yawning. Adult female JD (Green arrow) stopped grooming SBW; another adult (XY, Brown arrow) approaches SBW. Two other juveniles of the OMU (J1, Black arrow; J2, Light grey arrow) embrace while looking one from slightly farther away.

B: 06:12: Juvenile J3 (Blue arrow) gently mouths the face of the dead juvenile.

C: 13:01: The mother brings the dead juvenile’s head to her chest.

D: 13:03: The mother turns and corpse around and holds it as she walks clumsily.

E: 13:07: With the corpse’s face held to her chest, the mother carries/drags the dead juvenile while she walks.

F: 14:52: A juvenile (Bright green arrow) and an infant (Pale green arrow) from another unit approach, look, touch, and groom the dead juvenile, which the mother has temporarily left on the ground as she feeds nearby.

G: 17:09: An extra-unit juvenile (Violet arrow) approaches and watches as the mother holds her dead juvenile with both hands and emits calls (“Wa Wa Wa”).

H: 17:10: The mother holds the corpse with one hand and walks tripedally.

I: 17:10: The mother laid the body on the ground and emitted “Ah Ah Ah Ah” calls, possibly in response to the approach of people.

J: The mother half carries, half drags the corpse as she tries to walk.

K: 17:15: The OMU adult male calls toward the mother of the dead juvenile; in response she simply looks in the direction of the calls.

L: 06:10: The mother lags behind the rest of the group, lays the corpse on the ground.

M: 06:14: As the mother carries the corpse an extra-unit juvenile (Blue-grey arrow) approaches and looks from a tree.

N: 06:19: The mother carries the corpse and approaches her OMU. An extra-unit juvenile (Indigo arrow) approaches to look; an extra-unit adult female (Sky-blue arrow) and juvenile (Rose arrow) also look on.

O: 06:20 The mother picks up a few leaves and places them on the corpse; a juvenile (Teal arrow) approaches and briefly observes.

P: 06:21: The mother sits with the corpse, while an infant from the OMU (Dark-yellow arrow) approaches and looks.

Q: 06:21: The mother vocalizes as she sits alone with the corpse.

R: 06:45: With difficulty the mother carries the corpse into a tree, passing close by two adult females of another OMU, who watch (Gold arrow).

S: 06:50: The mother sits in a tree, the corpse draped over her lap and a branch.

T: 07:01: The corpse (Yellow arrow) gets left draped over a branch while the mother (Red arrow) moves nearer to her OMU’s male leader (White arrow).

U: 07:30: Having retrieved the corpse, the mother puts its head on the left breast, similar to breastfeeding behavior.

V: 17:21: The corpse is now clearly decaying, with fur missing in some places.

W: 17:21: The mother holds the corpse and tries to follow the group.

X: 17:21: The mother sits, having put the corpse on the ground, and looks in the direction of the group.

Y: 11:06: A clump of fur from the dead juvenile.

Z: 11:07: The corpse, now abandoned and maggot-infested.

a: 11:27: CM (Red arrow) sits alone in alone in tree and self-grooms, while alternating looks between the direction of the group (Blue arrow) and the abandoned corpse of her juvenile (Yellow arrow).

b: 11:56: CM (Red arrow) sits next to her OMU’s adult male (White arrow), with AF1 (Brown arrow) nearby.

c: 11:57: CM forages on the ground, near the OMU’s adult male.

d: 12:00: While still foraging, and alone, CM looks in the direction where she left the corpse, and emits contact calls (“Wa Wa Wa”).

e: 12:00: CM, sitting alone, looks in the direction of the rest of the group.

f: 12:10: CM approaches and starts to groom the OMU adult male, and a juvenile grooms CM.

g: 12:18: CM sits on the periphery of her OMU while the other members forage.

h: 12:18: Having moved to sit on a boulder, CM looks toward where she abandoned her juvenile’s corpse.

i: 12:18: CM continues to sit on a boulder and alternates between self-grooming and looking toward where she abandoned her juvenile’s corpse.

j: 12:13: Having approached each other, CM and the OMU adult male are sitting close to each other.

k: 12:13: CM grooms the OMU adult male.

l: 12:13: CM continues to groom the OMU adult male.

m: 12:56: Some of the monkeys notice a research assistant burying the abandoned corpse; they give occasional alarm calls from surrounding trees.

n: 12:40: The day after having abandoned her juvenile’s corpse, CM occasionally emitted contat calls (“Wa Wa “Wa”), for example, after foraging alone.
